# Coccydynia Treated with Dorsal Root Ganglion Stimulation

**DOI:** 10.1155/2018/5832401

**Published:** 2018-04-29

**Authors:** Nicholas L. Giordano, Noud van Helmond, Kenneth B. Chapman

**Affiliations:** ^1^Spine & Pain Institute of New York, New York City, NY, USA; ^2^Department of Anesthesiology, New York University Langone Medical Center, New York City, NY, USA; ^3^Northwell Health, New York City, NY, USA

## Abstract

Coccydynia can be difficult to resolve with conventional treatment options. Dorsal root ganglion (DRG) stimulation has recently emerged as a treatment for chronic pain, but its application has not been described in the context of coccydynia. We used DRG stimulation treatment in a patient suffering from intractable coccyx pain. At long-term follow-up, the patient experienced a decrease in pain intensity and improvement in function, without any complications. DRG stimulation may be a treatment modality for coccydynia refractory to other approaches.

## 1. Introduction

Coccydynia, or coccygodynia, is pain in the region of the coccyx. The term was first coined in 1859 by Simpson [1], who also introduced the use of chloroform in anesthesia. Despite the identification of coccygeal pain almost ~150 years ago, its treatment can be difficult in a select patient population in whom the pain becomes chronic and debilitating. Treatment options range from lifestyle modification and cushions, to injections, to treatments as radical as resection of the coccyx [2]. Recent reports have described the use of neuromodulation techniques to treat chronic coccydynia [3]. The aim of this report is to describe the use of dorsal root ganglion (DRG) stimulation to treat chronic coccydynia.

Written informed consent was obtained from the patient for publication of this case report.

## 2. Case Description

A 37-year-old female from Spain presented to our center in August of 2017 with complaints of chronic intractable pain in the coccyx. The patient suffered from this pain since 2009 when she sustained a coccyx fracture in a work-related slip and fall injury while working as an airline stewardess in Europe. At the time of presentation, the patient endorsed 10/10 coccyx pain on visual analog scale. She described the pain as sharp, stinging, shooting, and radiating throughout her bilateral lower extremities. Due to the injury, she was forced to quit when her pain prevented her from performing her duties. At the time of presentation, she could not sit or walk for more than 10 minutes consecutively and required special cushioning to be brought with her at all times.

Prior to presenting to our center, she had been evaluated and treated by pain management physicians, orthopedic surgeons, and multiple urgent care clinicians in both Spain and the UK. Her past treatments in Europe included multiple coccygeal blocks, trigger point injections, epidural steroid injections, and a conventional spinal cord stimulator in 2011. The spinal cord stimulation therapy consisted of an ANS Genesis (company acquired in 2005 by St. Jude Medical, which was subsequently purchased by Abbot) spinal cord stimulator with one octode lead placed through the sacrococcygeal hiatus. This stimulator was placed in Spain in 2011 and the patient continued to use the stimulator at presentation. Stimulator treatment was previously complicated by an infection, which was treated with IV antibiotics and surgical debridement. The patient reported pain relief from the stimulator, but she was experiencing diminished relief from the stimulator over the last several years and also had inadequate coverage in her most painful region, which was the coccyx itself. She continued to have the stimulator turned on as it provided some relief, but she was still incapacitated from the pain. She was maintained on a pain medication regiment consisting of oxycodone 10 mg PO BID, dexketoprofen 25 mg PO QID, duloxetine 60 mg PO QD, trazodone 100 mg PO QD, and pregabalin 75 mg PO BID. Despite this intensive medical treatment, the patient experienced poor symptom control in addition to side effects from these medications, including constipation and drowsiness.

The patient presented to us seeking other potential options for pain relief, in particular a conventional radiofrequency ablation of the nerves innervating the coccyx or an endoscopic radiofrequency ablation of those nerves. Considering the patient's persistent severe coccydynia and failure of extensive conservative and interventional treatments and the chronicity of her pain, we proposed a DRG stimulator trial in September of 2017. The use of DRG stimulation for coccydynia is an off-label use; the reason we considered DRG stimulation was secondary to our highly successful experiences with DRG stimulation for both complex pelvic and rectal pain, as well as low back and SI joint pain. Our rationale for proceeding with the trial was the potential for better regional coverage versus conventional sacral nerve stimulation, similar to our experience in those cases.

Our proposed approach was a bilateral L1 and S2 DRG stimulator trial. The patient decided to proceed with the DRG stimulator trial and underwent psychological clearance prior to the procedure. During the 7-day trial, the patient rated her pain less than 1/10 on visual analog score, improved sleep hygiene, functioned better in general, and was actually able to ambulate for approximately four miles without limitations. Prior to the trial she could not walk more than a city block without severe pain. She was able to function better in almost all her daily activities and she was able to position herself with minimal limitations and without the aid of her cushion, which she previously carried with her at all times, and claimed to have close to 100% coverage of her pain. After the trial, the patient decided to proceed with permanent lead implantation. She understood she had the option to have the procedure performed in Europe or to have it performed at our institute. After consideration, she decided to proceed with the implantation at our center. We then decided to leave her current spinal cord stimulator system in place rather than to explant the system.

### 2.1. Surgical Procedure

**Figure 1:**
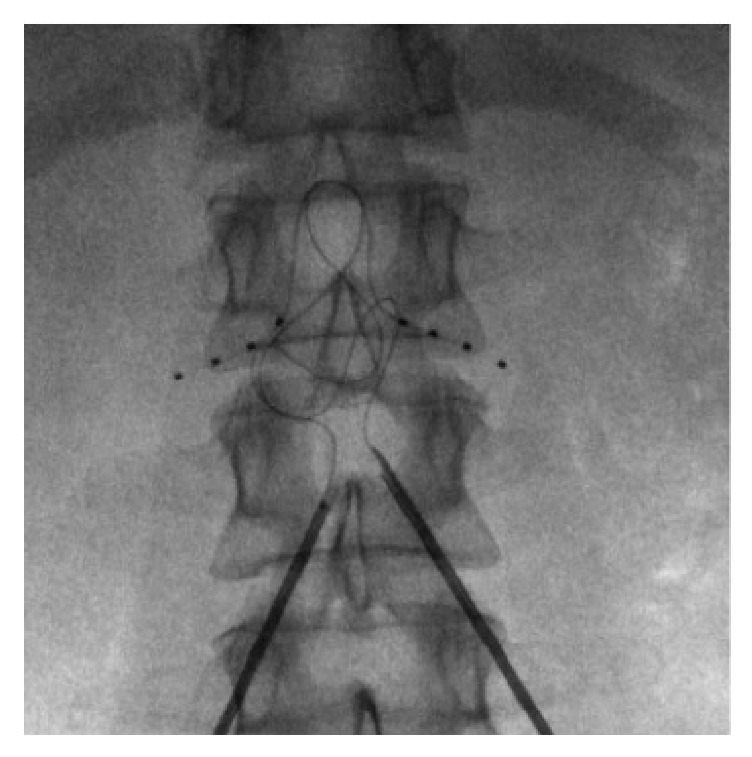
Anterior posterior fluoroscopic image of bilateral dorsal root ganglion stimulation leads on the L1 level.

**Figure 2:**
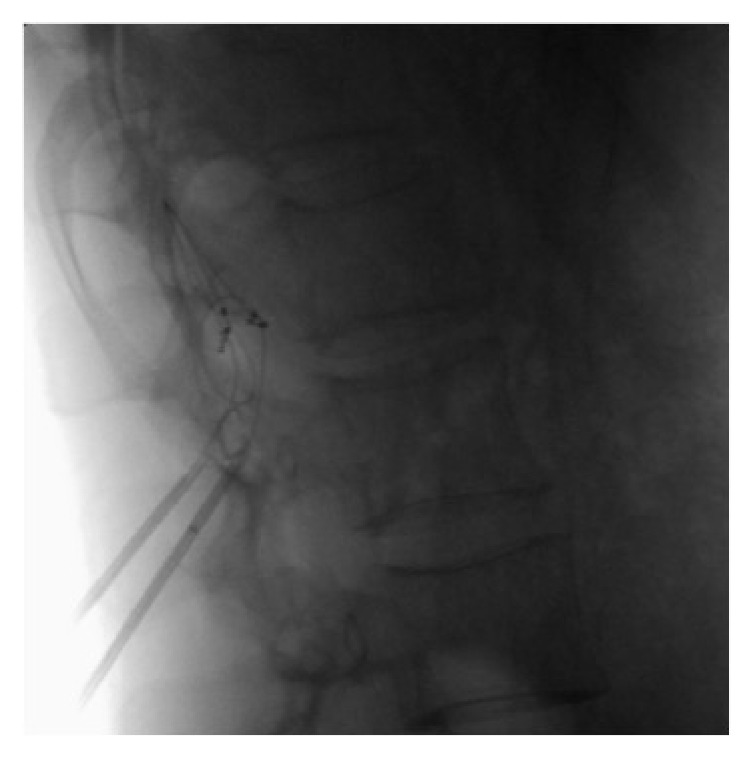
Lateral fluoroscopic image of bilateral dorsal root ganglion stimulation leads on the L1 level.

A board-certified anesthesiologist monitored the patient throughout the implant procedure. Thirty minutes prior to the procedure the patient received 2 grams of cefazolin. The patient was placed in the prone position with bolsters under the lumbar/lower thoracic region. After positioning was deemed adequate, the patient received propofol sedation. The lumbar spine and buttocks were prepped and draped in normal sterile fashion with betadine followed by DuraPrep. The L1 vertebral body was then aligned on fluoroscopy and 1.0% lidocaine mixed with 0.5% bupivacaine was used for local anesthesia. A Tuohy needle was inserted at the right side one level below the left L1 target foramen at the level of the pedicle and was then guided toward the midline at the interspace of the target level. Loss of resistance to air was achieved close to the midline. At this point, the 4-contact DRG lead (Axium™, St. Jude/Abbot, Lake Bluff, IL) was loaded into the introducer catheter with the lead tip approximately 2-3 mm outside of the introducer. The loaded introducer was then passed into the Tuohy needle. The introducer then accessed the epidural space and was directed toward the foramen. The introducer was passed through the target foramen until the middle two contacts were under the level of the pedicle. The introducer was then withdrawn to approximately 5 mm after the proximal contact while applying counterforce on the lead. A strain relief loop was created in the usual fashion [4]. Subsequently, the introducer was removed from the Tuohy needle and the lead was left in place. Fluoroscopy was performed to confirm no displacement of the lead had occurred. The same procedure was performed to place a lead on the right L1 DRG. Figures 1 and 2 depict the position of bilateral leads on the L1 level.

**Figure 3:**
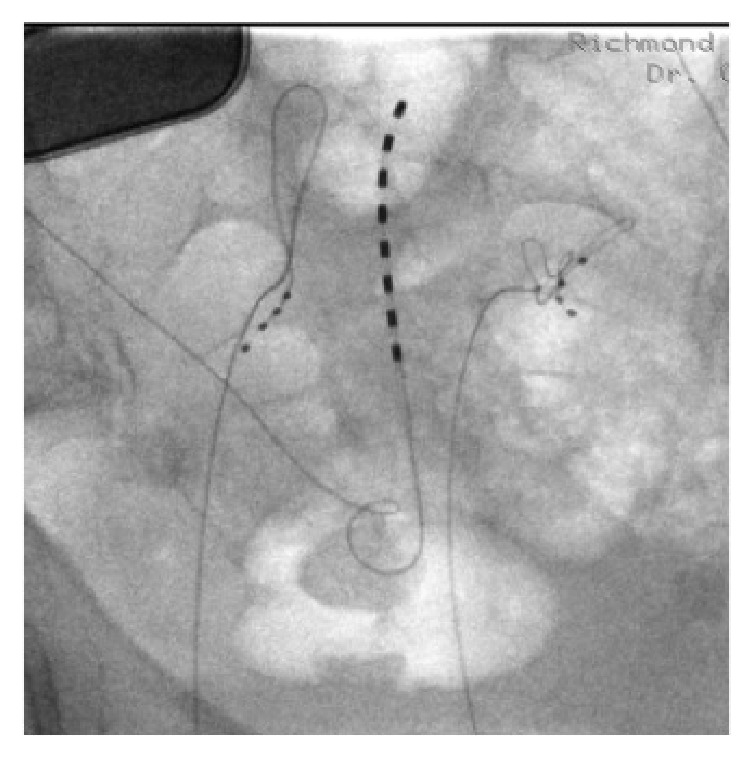
Anterior posterior fluoroscopic image of bilateral dorsal root ganglion stimulation leads on the S2 level. The trans-sacral-hiatus octode lead and battery for her previous conventional sacral neuromodulation therapy can be appreciated as well.

**Figure 4:**
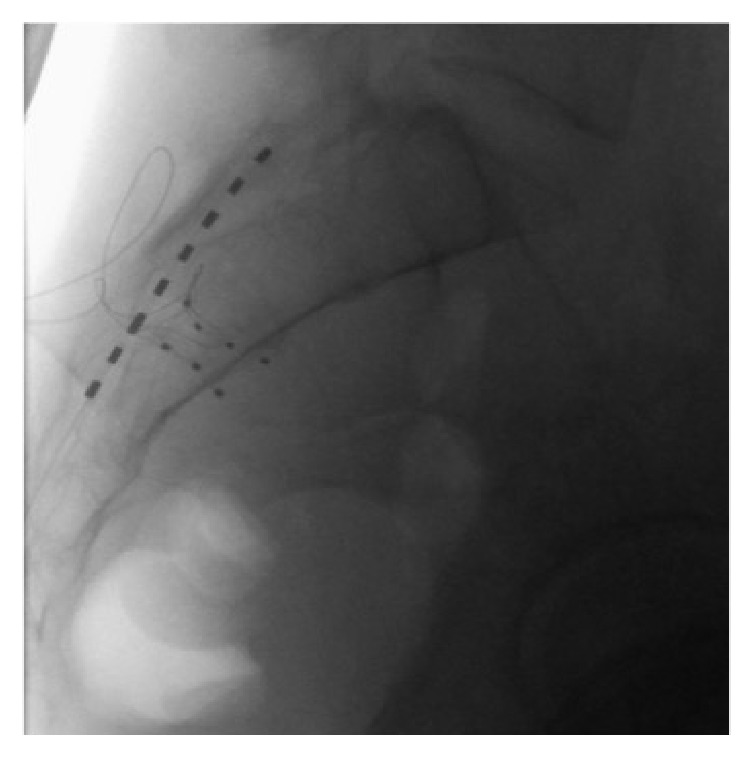
Lateral fluoroscopic image of bilateral dorsal root ganglion stimulation leads on the S2 level. The trans-sacral-hiatus octode lead for her previous conventional sacral neuromodulation therapy can be appreciated as well.

At this point the right S2 foramen was aligned under fluoroscopy. A Tuohy needle was directed into the posterior S2 foramen and its position was confirmed on fluoroscopy. The lead loaded introducer was then passed through the anterior foramen and the electrodes were maneuvered such that the final position was with 1 contact anterior to the anterior wall of the sacral vertebral body (extraforaminal), 2 contacts intrasacral, and 1 contact in the sacral epidural space. A strain relief loop was created as previously described [4]. The introducer was then withdrawn into the Tuohy needle with the lead left in place. In withdrawing the Tuohy needle another small loop was placed subcutaneously for additional tensile strength. The lead position was checked again in the AP and lateral position. The same procedure was performed to place a lead on the left S2 DRG. Figures 3 and 4 depict the position of bilateral leads on the S2 level.

All 4 lead positions were then checked again and found to be in good position as well as their tension loops. The leads were then secured with Tegaderm. A marking was placed on the right buttock that was 4 cm transverse in the upper outer quadrant, since the patient's ANS Genesis stimulator was implanted on the left side. 10 cc of 1.0% lidocaine mixed with 0.5% bupivacaine was used to anesthetize the incision site. An incision was made and a pocket was created. The leads were tunneled to the pocket as previously described [5]. The leads were connected to the pulse generator and impedances were confirmed. The incisions were irrigated with Bacitracin solution and the generator was anchored with 2.0 Ethibond. The right buttock wound was closed in layers by using 2.0 Vicryl and the skin was closed with staples. All 4 lead puncture sites were small and the leads were not visualized with manipulation of the puncture site. The lead placement puncture sites and the buttock incision were covered with Steristrips followed by gauze and Tegaderm.

On four-month follow-up, the patient still reports >90% pain relief from the stimulator therapy with concomitant improvements in daily functioning. The improvement in pain control allowed her to discontinue her oxycodone, which was causing her to suffer from side effects before.

## 3. Discussion

The aim of this report was to describe the successful application of DRG stimulation for chronic coccydynia. Coccydynia is prevalent and can be difficult to treat in chronic cases. Factors associated with increased risk of developing coccydynia include obesity and female gender [6]; women are 5 times more likely to develop coccydynia than men. The most common etiology of coccydynia is trauma, consistent with the presented case. External trauma usually occurs due to a backwards fall, leading to a bruised, dislocated, or broken coccyx [7]. Patients typically present complaining of “tailbone pain.” The pain will usually be worse with prolonged sitting, leaning back while seated, prolonged standing, and rising from a seated position. X-ray and magnetic resonance imaging can be used to evaluate for the presence of fractures, degenerative changes, or masses. Most cases of coccydynia resolve within weeks to months with or without conservative treatment [8]. Conservative treatment consists of cushions, the application of heat and cold, nonsteroidal anti-inflammatory drugs, and transcutaneous electrical nerve stimulation.

For the few cases that do not respond to these conservative treatments, more aggressive treatments may be indicated. Injections around the coccyx, usually at the sacrococcygeal junction or around the sacrococcygeal ligaments, of local anesthetic with steroid can be both diagnostic and therapeutic [9]. Another approach is to target the ganglion impar, also known as the ganglion of Walther [10]. The ganglion impar is the pelvic portion of the sympathetic trunk located in the midline anterior to the sacrococcygeal junction. This block can be useful in refractory cases and cases associated with pelvic pain, as well as for pain associated with malignant neoplasms. Surgical procedures for the treatment of coccydynia have been reported consisting of surgical amputation of the coccyx just proximal to the sacrococcygeal junction [11]. However, this procedure may be associated with a high complication rate and failure to relieve the pain. Consequently, based on current available information, this procedure generally is not recommended [2].

Recent case reports support the use of different neuromodulation techniques to treat refractory coccydynia. In 2008 Haider [12] reported on the use of conventional spinal cord stimulation to successfully treat a patient with chronic coccydynia. Another report by Vajramani et al. [13] described the successful use of high frequency 10 kHz spinal cord stimulation to the conus/cauda region in two patients with chronic coccydynia. Our report adds to this by describing the successful application of DRG stimulation for coccydynia. The DRG has been of interest to pain physicians for years since scientific evidence on spinal structures suggests that the DRG is an integral part of both nociceptive and neuropathic pain states [14]. The suggested mechanism of electrical stimulation of the DRG is a reduction of action potential conduction at the bifurcation (T-junction) of sensory neurons within the DRG, resulting in the reduction of perceived pain [15]. Most studies to date have focused on DRG stimulation for neuropathic pain states [14]. Both neuropathic and nociceptive components are believed to contribute to chronic coccydynia [3], and the instrumental role of the DRG in both pain states may explain the positive results achieved in the present case.

### 3.1. Conclusion

We successfully used DRG stimulation to treat chronic coccydynia. Future studies need to corroborate the effectiveness of DRG stimulation for this indication.
